# HIV-1 Vpr protein upregulates microRNA-210-5p expression to induce G_2_ arrest by targeting TGIF2

**DOI:** 10.1371/journal.pone.0261971

**Published:** 2021-12-29

**Authors:** Jialu Qiao, Qian Peng, Feng Qian, Qiang You, Lingyan Feng, Song Hu, Wei Liu, Lixia Huang, Xiji Shu, Binlian Sun

**Affiliations:** 1 Wuhan Institute of Biomedical Sciences, School of Medicine, Jianghan University, Wuhan, China; 2 Division of HIV/AIDS, The Second Affiliated Hospital of Soochow University, Soochow, China; 3 Department of Immunology, School of Medicine, Jianghan University, Wuhan, China; Institute of Human Virology, UNITED STATES

## Abstract

MicroRNAs (miRNAs) are important molecules that mediate virus-host interactions, mainly by regulating gene expression via gene silencing. Here, we demonstrated that HIV-1 infection upregulated miR-210-5p in HIV-1-inoculated cell lines and in the serum of HIV-1-infected individuals. Luciferase reporter assays and western blotting confirmed that a target protein of miR-210-5p, TGIF2, is regulated by HIV-1 infection. Furthermore, HIV-1 Vpr protein induced miR-210-5p expression. The use of a miR-210-5p inhibitor and TGIF2 overexpression showed that Vpr upregulated miR-210-5p and thereby downregulated TGIF2, which might be one of the mechanisms used by Vpr to induce G2 arrest. Moreover, we identified a transcription factor, NF-κB p50, which upregulated miR-210-5p in response to Vpr protein. In conclusion, we identified a mechanism whereby miR-210-5p, which is induced upon HIV-1 infection, targets TGIF2. This pathway was initiated by Vpr protein activating NF-κB p50, which promoted G2 arrest. These alterations orchestrated by miRNA provide new evidence on how HIV-1 interacts with its host during infection and increase our understanding of the mechanism by which Vpr regulates the cell cycle.

## Introduction

By the end of 2020, there were around 40 million people worldwide who were infected with HIV-1, the causative agent of AIDS. HIV-1 can weaken a person’s immune system by attacking CD4^+^ T cells and macrophages, making the person more vulnerable to opportunistic infections. Although the success of highly antiretroviral therapy (ART) has turned the once-lethal disease into a chronic infection, HIV-1 remains incurable and the virus can become latent and then reactivate at later points. HIV-1 can rapidly develop resistance to therapy, evade the immune response, alter cell proliferation, and induce apoptosis of infected cells [1, 2]. A better understanding of the interactions between HIV-1 and its host cells may promote the development of AIDS treatments.

HIV-1 infection involves numerous competing and complementary interactions among viral and cellular proteins. On the one hand, signaling pathways in mammalian cells can be activated to induce a variety of intracellular events in infected cells to defend against virus replication. On the other hand, the virus can escape the host defenses by interacting with components of the infected cell and subverting the cell proliferation, signal transduction, and transcriptional machineries [3]. As an accessory protein of HIV-1, Vpr has multiple functions in HIV-1-infected cells, including inducing arrest at the G2 phase of cell division and modulating cell signaling [4]. Many studies have explored the mechanism of Vpr-induced G2 arrest, which involves Vpr-induced activation of ATR and Chk1 kinases and inactivation of the cyclinB-Cdk1 complex [5]. Other viral factors, such as Wee1, the phosphatase PP2A, and Rad24, are also involved in Vpr-mediated cell cycle arrest [6]. Additionally, Vpr-binding protein (VprBP) has been recognized as a critical host factor in the ability of Vpr to trigger cell cycle arrest. Vpr recruits the DDB1 and Cul4A containing ubiquitin-ligase complex via VprBP and the substrates of ubiquitin-ligase complex are then ubiquitinated and degraded to trigger G2 arrest [7, 8]. Recently, research has shown that Vpr protein also interacts with SLX4 via its SLX1-binding domain, resulting in G2 arrest [9]. In summary, Vpr can induce G2 arrest by affecting host proteins that maintain the cell cycle, and Vpr affects cell division via a number of pathways.

MicroRNAs (miRNAs) are a class of small non-coding RNAs that can act via RNA interference to reduce protein expression, thereby post-transcriptionally regulating target gene expression. These molecules govern a wide range of biological functions including cell differentiation, proliferation, and apoptosis [10]. They are also involved in host-virus interactions, including controlling virus replication and evading the host antiviral response by regulating host genes [11]. Research has identified miRNA profiles regulated by HIV-1 infection and the effects of these miRNAs on HIV-1 replication and pathogenesis [12]. Swaminathan et al. reported that HIV-1-induced miR-155 upregulation is associated with decreased HIV-1 infectivity in macrophages [13]. Chen et al. showed that miR-146a inhibited Gag assembly and virus production by binding to HIV-1 Gag [14]. miR-29a and 29b are expressed in human peripheral blood mononuclear cells, resulting in repression of the target Nef protein and reduction of virus levels [15, 16]. Tat induces miR-34a and miR-138 expression to downregulate SIRT1 and promote astrocyte activation [17]. These studies indicate that exploring the roles of miRNAs during HIV-1 infection can have profound impact on our understanding of the mechanisms underlying HIV-1 infection.

To further study the miRNA-mediated interactions between HIV-1-and its host cells, we performed microarray analysis and qRT-PCR detection using HIV-1-inoculated MT4 cells. The results revealed that HIV-1 infection upregulated several miRNAs, particularly miR-210-5p. We found that a target gene of miR-210-5p is TGIF2 (transforming growth factor-beta-induced 2), a member of the TALE (three-amino-acid loop extension) superfamily [18, 19]. TGIF2 is a transcription regulator that plays essential roles in the regulation of development and cell fate decisions. Increased TGIF2 protein levels have been implicated in many cancers, including ovarian, esophageal, lung, and colon cancer, which implies that TGIF2 might be involved in cell proliferation [20–23]. In melanoma, TGIF2 directly upregulates the transcription of FUT8 (fucosyltransferase 8) to induce metastasis, increasing the aggressive nature of the cancer [24]. Further experiments showed that the HIV-1 Vpr protein triggers TGIF2 downregulation via miR-210-5p. Based on the various functions of Vpr, we speculate that enhancing miR-210-5p to downregulate TGIF2 is one mechanism of HIV-1 Vpr-induced G2 arrest. This study extended our knowledge of the interactions between viruses and host cells involving miRNA.

## Methods

### Ethics statement

All research involving human participants was approved by the Institutional Review Board of Soochow University in accordance with their guidelines for the protection of human subjects and was conducted in accordance with the provisions of the Declaration of Helsinki. Approval was obtained from the Second Affiliated Hospital of Soochow University, China (approval number: 2018003). Written informed consent was obtained from each participant.

### Participant recruitment

From May 2018 to December 2019, 20 adult patients who received HIV-1 treatment at the Second Affiliated Hospital of Soochow University were included in the study. These patients had confirmed HIV infection, no hepatitis C-related liver diseases, no autoimmune diseases, and were not taking antibiotics or antiretrovirals. Additionally, 20 sex- and age-matched healthy volunteers with negative markers for HIV-1 served as controls. Following blood sample collection, the samples were centrifuged and the serum was stored at −70°C.

### Cell culture and antibodies

Human MT4 T cells and Jurkat cells were grown in Roswell Park Memorial Institute (RPMI) 1640 (Gibco, Logan, UT, USA) at 37°C in 5% CO_2_. HOS.CD4.CCR5 cells (hereinafter referred to as HOS cells) and 293T cells were grown in complete Dulbecco’s Modified Eagle’s Medium (DMEM; Gibco, Logan, UT, USA) supplemented with 10% heat-inactivated fetal calf serum at 37°C in 5% CO_2_. The cells were subsequently transfected using Lipofectamine 2000 (Invitrogen, Carlsbad, CA, USA) according to the manufacturer’s instructions or inoculated with HIV-1 as described previously [25]. All cells were purchased from the Cell Bank of the Chinese Academy of Science (Shanghai, China).

Antibodies against HIV-1 p24 (sc-69728) and phosphor-p50 (p-p50; sc-271908) were purchased from Santa Cruz Biotechnology (Santa Cruz, CA, USA). Antibodies against p50 (14220-1-AP), Actin (66009-1-Ig), LaminB (12987-1-AP), and Flag (80010-1-RR) were purchased from Proteintech (Rosemont, IL, USA). Antibody against TGIF2 (ab155948) was purchased from Abcam (Cambridge, MA, USA). Antibody against Vpr (MA-001-2) was purchased from Cosmo Bio Co., Ltd. (Tokyo, Japan).

### Plasmid construction

The primers used for plasmid construction are listed in S1 Table. The full-length TGIF2 and its 3`untranslated region (UTR) were amplified from cDNA in HOS cells using corresponding primers, and the PCR fragments were cloned into pCMV-Flag expression vectors. Both the full-length and a series of truncated promoters of miR-210-5p were amplified from 293T genomic DNA using corresponding primers, and the fragments were then digested by Kpn I and Hind III and cloned into pGL3-basic. Mutated miR-210-5p promoters were generated by site-directed mutagenesis using specific primers. A TGIF2 3’ UTR luciferase reporter construct was generated by cloning PCR-amplified human TGIF2 mRNA 3’ UTR target sites into the Sac I and Sal I sites of pmirGLO.

HIV-1 pNL4-3.Luc.R+E- or pNL4-3.Luc.R-E- plasmids, containing the firefly luciferase gene in the nef position and the Env-inactivating mutation [26], were provided by Dr. K. Tokunaga (National Institute of Infectious Diseases, Tokyo, Japan). The former contains the full-length vpr gene, while the latter cannot express Vpr protein because of a frameshift in the vpr gene. pNL4-3.GFP.R+E- and pNL4-3.GFP.R−E- plasmids were derived from the abovementioned plasmids, respectively, by replacing the firefly luciferase gene with the enhanced green fluorescent protein (EGFP) gene.

### Single-cycle infectivity assay

The HIV-1 pNL4-3.Luc.R+E- or pNL4-3.Luc.R−E- constructs and HIV-1 pNL4-3.GFP.R+E- or pNL4-3.GFP.R−E- constructs were pseudotyped with vesicular stomatitis virus envelope glycoprotein (VSV-G) and denoted HIV-1 or HIV-1-GFP (with functional Vpr) or HIV-1-Vpr^-^ or HIV-1-GFP-Vpr^-^ (with Vpr deleted), respectively. Briefly, 293T cells (2×10^6^) were seeded in 10-cm culture dishes; the following day, the cells were co-transfected with 7.5 μg of above HIV-1 constructs and 2.5 μg of VSV-G expression plasmid using Lipofectamine^TM^2000 Transfection Reagent (Invitrogen, Carlsbad, CA, USA). The culture medium was changed 4–6 h after transfection, and the virus-containing supernatants were collected 48 h later. The supernatants were filtered through a 0.45-μm filter to remove cellular debris, divided into aliquots, and stored at −80°C for the inoculation assays. The viral titers were measured with a p24 ELISA kit (Advanced BioScience Laboratories, Inc,USA). Cells were inoculated with pseudotyped HIV-1 for 36 h and 100 ng/ml of p24^gag^ unless stated otherwise.

### Microarray assay of miRNA

Total RNA was harvested from HIV-1-GFP-inoculated (100 ng/mL) and mock-treated MT4 cells (after inoculation for 48 h) using TRIzol (Invitrogen, Carlsbad, CA, USA) and miRNeasy Mini Kit (Qiagen, Dusseldorf, Germany) according to the manufacturer’s instructions. After assessing the RNA quantity using a NanoDrop 1000 spectrophotometer (NanoDrop Technologies, Wilmington, DE, USA), the samples were labeled using a miRCURY™ Hy3™/Hy5™ Power labeling kit and hybridized on a miRCURY™ LNA Array (v.16.0). Following the washing steps, the slides were scanned using a GenePix 4000B microarray scanner (Axon Instruments, Union City, CA, USA). The scanned images were then imported into GenePix Pro 6.0 software (Axon Instruments, Union City, CA, USA) for grid alignment and data extraction. Replicated miRNAs were averaged and miRNAs with intensities ≥50 in all samples were selected to calculate the normalization factor. Expression data were normalized using median normalization. Thereafter, significantly differentially expressed miRNAs between HIV-1-inoculated and mock-treatted cells were identified using volcano plot filtering. Finally, hierarchical clustering was performed to visualize the different miRNA expression profiles between the groups (fold change ≥1.5; P≤0.05). The Gene Expression Omnibus (GEO) accession number for these miRNA-seq data is GSE161444.

### RNA quantification

Total RNA was extracted from the cells using TRIzol reagent (Invitrogen, Carlsbad, CA, USA) according to the manufacturer’s protocol, and it was then reverse transcribed using M-MLV Reverse Transcriptase (Invitrogen, Carlsbad, CA, USA). Additionally, RNA was extracted from the serum of HIV-1-infected and healthy individuals using RNA kits (Roche, IN, USA) according to the manufacturer’s instructions.

Quantification of miRNAs was performed by qPCR using miRNA analysis kits (Applied Biosystems, Carlsbad, CA, USA) according to the manufacturer’s instructions. The primers for the miRNAs were purchased from GuangZhou RiBo Biotech Co. Ltd. (GuangZhou, China). The relative expression of the miRNAs was normalized to that of U6 snRNA (internal control) using the 2^−ΔΔCt^ method.

### miRNA mimics and inhibitors and controls

miR-210-5p mimics (double-stranded RNA oligonucleotides) and miR-210-5p inhibitors (single-stranded chemically modified oligonucleotides) (GuangZhou RiBo Biotech Co. Ltd., GuangZhou, China) were transfected into cells for miR-210-5p overexpression and inhibition, respectively. Nonspecific RNA mimics or inhibitors were transfected as negative controls (NC). All oligonucleotide sequences are listed in S1 Table.

### Luciferase reporter assays

Luciferase activity was assayed using a Luciferase Reporter Assay System (Promega, Madison, WI, USA) according to the manufacturer’s instructions. Briefly, HOS cells were co-transfected with the luciferase reporter plasmid and the relevant RNAs. After 24 h, the cells were harvested, washed with phosphate-buffered saline (PBS), transferred to a 24-well plate, and 100μL of lysis buffer was added to each well. Next, 20 μL of the lysed cells was mixed with the luciferase assay substrate. Regarding the single luciferase reporter assays, the firefly or *Renilla* luciferase activity was normalized based on the optical density of each sample. Regarding the dual-luciferase reporter assays, first, firefly luciferase activity was normalized based on the activity of *Renilla* luciferase. Thereafter, the quantitative value in each construct was normalized to the control.

### Western blot analysis

Whole-cell lysates were prepared by lysing cells with PBS (pH 7.4) containing 0.01% Triton-100, 0.01% ethylenediaminetetraacetic acid (EDTA), and 10% cocktail protease inhibitor (Roche, Basel, Switzerland). The protein concentration was determined by the Bradford assay (Bio-Rad, Redmond, WA, USA). Cell lysates were separated by 10% sodium dodecyl sulfate polyacrylamide gel electrophoresis (SDS-PAGE) and then transferred to Immobilon-P membranes (Merck Millipore Ltd., Darmstadt, Germany). The membranes were blocked with 5% nonfat dried milk before incubating them with target-specific primary antibodies followed by horseradish peroxidase (HRP)-conjugated secondary antibodies. The bands were visualized using a ChemiDoc™ XRS+ system (Bio-Rad Laboratories, Inc., CA, USA) after incubating each membrane with Western Bright Sirius HRP substrate (Millipore, MA, USA). Quantification was performed using Quantity One 1-D Analysis software (Bio-Rad Laboratories, Inc., CA, USA).

Additionally, as NF-κB p50 regulates gene transcription when it moves to the nucleus, we assessed p50 expression in the nucleus and cytoplasm in HIV-1-inoculated MT4 cells compared to in mock-inoculated cells or cells inoculated with HIV-1 Vpr^-^. Nuclear and cytoplasmic separation was performed using a separation kit (Beyotime Biotechnology, Beijing, China) according to the manufacturer’s instructions.

### Chromatin immunoprecipitation (ChIP) assays

ChIP assays were performed using reagents from Sigma-Aldrich (St. Louis, MO, USA), according to the manufacturer’s instructions with the following minor modifications. HOS cells were cross-linked in 1% formaldehyde for 10 min at room temperature. After washing twice with PBS containing protease inhibitors, the cells were lysed with 200 μL of SDS lysis buffer and the chromatin/DNA was sonicated to around 500 bp. Immunoprecipitation was performed by incubating the sheared chromatin (2×10^6^ cells) overnight at 4°C with 2 μg of antibody followed by binding to 60μL of Salmon Sperm DNA/ Protein G Sepharose beads. The immunopreciptates were washed, eluted in 1% SDS with 0.1M NaHCO_3_, and incubated overnight at 65°C with 20 μL of 5M NaCl for reverse cross-linking. The DNA was purified using QIAquick columns (Qiagen, Valencia, CA, USA) and each fragment was PCR-amplified using the primer pairs listed in S1 Table.

### Cell cycle assay

Cells were detached by incubation with trypsin and fixed with 70% ethanol at 4°C overnight, washed with PBS, and reacted with propidium iodide (PI, 20 μg/mL) for 30 min in the dark. The cells were then subjected to cell cycle distribution analysis using a flow cytometer (BD Biosciences, San Jose, CA, USA). Ten thousand cells were assessed using CellQuest Pro software (BD Biosciences, San Jose, CA, USA). The ratio of G2+M/G1 phase cells was calculated using ModFit LT 5.0 software (Verity Software House, Topsham, ME, USA).

### Statistical analysis

Each experiment was repeated three times. The expression data are presented as mean ± standard deviation. The mean of three experiments was compared between groups by one-way analysis of variance (ANOVA) with post-hoc Tukey’s test for multiple testing correction. The statistical analyses were performed using SPSS 22 (IBM Corp., Armonk, NY, USA). Significance was defined as *P*<0.05.

## Results

### miR-210-5p expression is induced by HIV-1 infection

**Fig 1:**
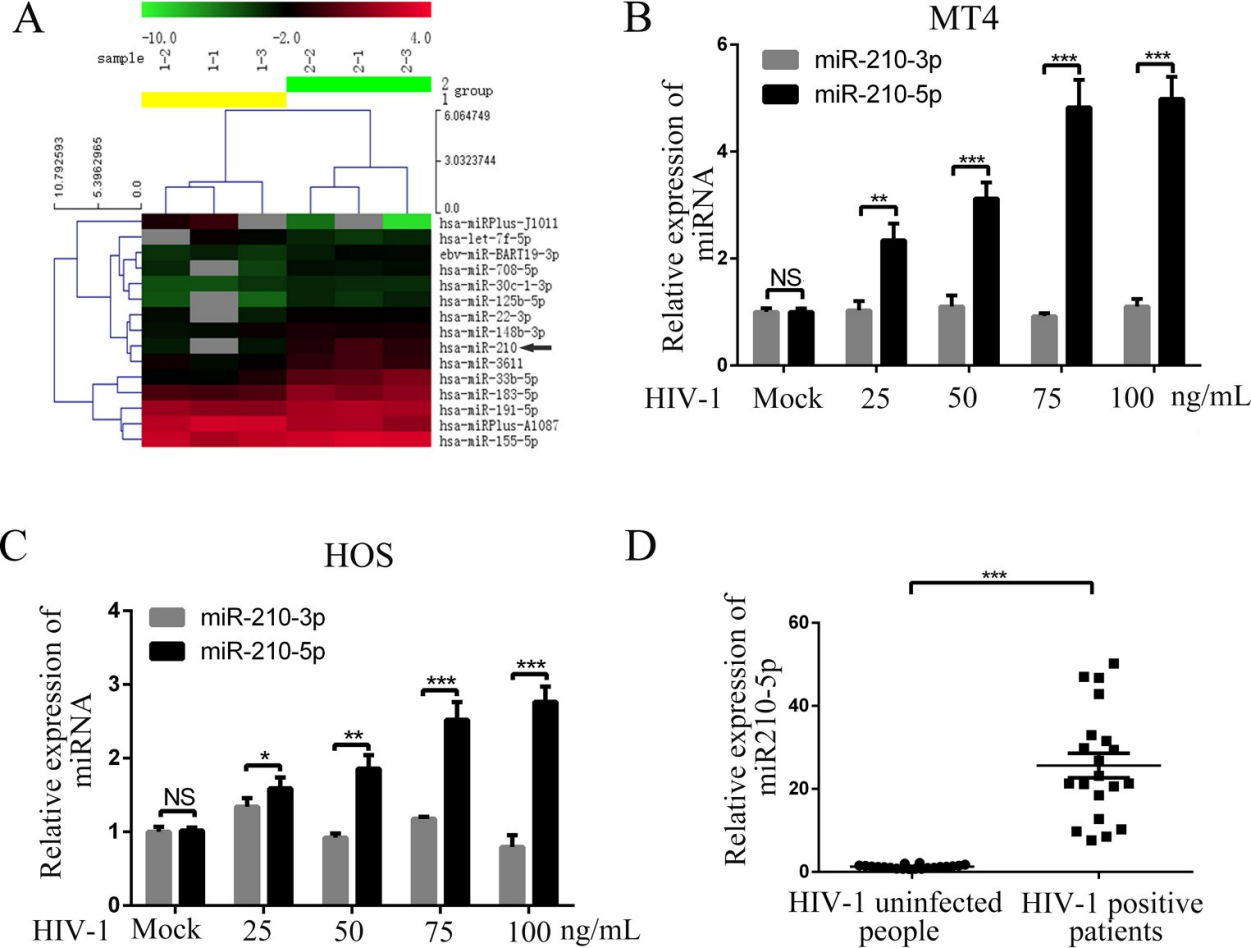
Determination of miR-210-5p expression during HIV-1 infection. (A) Heatmap of the relative miRNA expression in mock-inoculated MT4 cells (group 1) or MT4 cells inoculated with pseudotyped HIV-1-GFP (group 2). Arrow indicates miR-210. (B-C) MT4 and HOS cells were inoculated with HIV-1 for 48 h at various concentrations. miR-210-3p and miR-210-5p expression was measured with qPCR. (D) miR-210-5p levels in serum of HIV-1infected individuals and HIV-1- uninfected individuals were measured with qPCR. miR-210-5p expression in all experiments was normalized to U6 expression in each sample. The data are presented as mean ± SD for three biological replicates, and statistical significance compared to the controls was calculated by *t* test. **P < 0.01, ***P < 0.001, NS: not significant.

To investigate which miRNAs are regulated by HIV-1 challenge, we analyzed the miRNA expression profile of MT4 cells inoculated with HIV-1-GFP at 100 ng/mL for 48 h using array-based miRNA profiling. Flow cytometry indicated that >90% of the MT4 cells were inoculated with HIV-1. The miRNA array results revealed the differentially expressed miRNAs between the HIV-1-inoculated and mock-inoculated cells (fold change ≥1.5, P≤0.05). As shown in the heatmap (Fig 1A), there were 12 upregulated and 3 downregulated miRNAs and the most upregulated miRNA was miR-210 (S2 Table). Moreover, we detected the 11 changed miRNA in MT4 after HIV-1 infection by qPCR and found that miR-210 was also the most upregulated miRNA (S1 Fig). We focused on this miRNA to investigate the interactions between HIV-1 and host cells involving miRNA. As miR-210 generates two mature miRNAs, miR-210-3p (miRBase: MIMAT0000267) and miR-210-5p (miRBase: MIMAT0026475), we identified which form was upregulated in MT4 cells inoculated with increasing concentrations of HIV-1. The qPCR results showed that miR-210-5p levels were increased in the MT4 cells in a virus dose-dependent manner (Fig 1B). Similar results were observed in HOS cells (Fig 1C), which showed that miR-210-5p upregulation is a common characteristic in HIV-1 infection. We also assayed the expression level of miR-210-5p in Jurkat cells upon HIV-1 infection (S2 Fig). The result showed that miR-210-5p levels were also increased in Jurkat cells in a virus dose-dependent manner. In addition, we wondered whether the miR-210-5p level in the serum of HIV-1-infected patients was increased. The qPCR results indicated that the miR-210-5p level was significantly higher in HIV-1-infected individuals compared to uninfected individuals (Fig 1D). These *in vivo* observations further confirmed the results of cell culture, indicating that HIV-1 infection induces miR-210-5p expression.

### TGIF2 is a target of miR-210-5p

**Fig 2:**
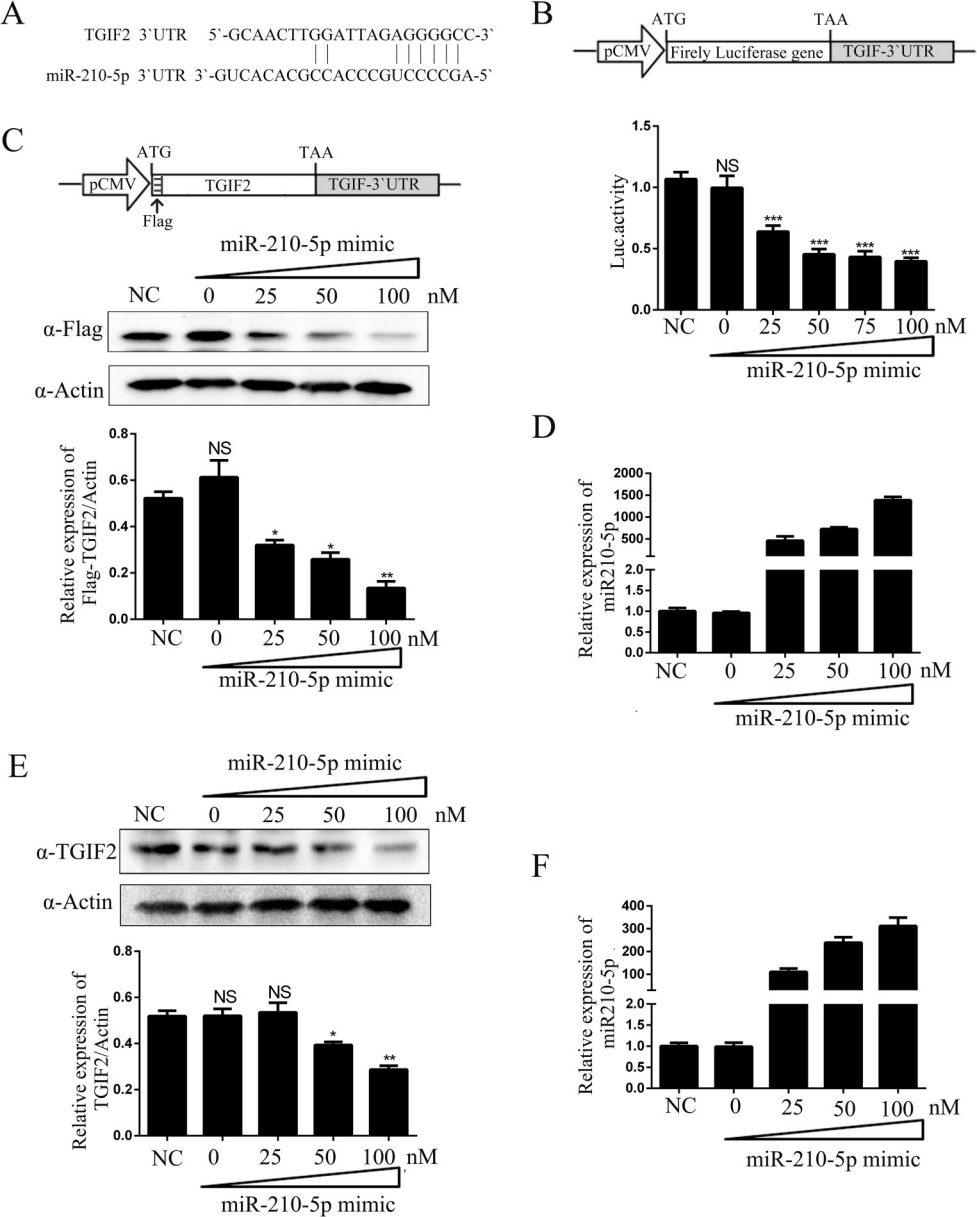
TGIF2 expression in miR-210-5p-overexpressing cells. (A) Predicted miR-210-5p binding sites in the TGIF2-3`UTR. (B) HOS cells were transfected with the luciferase reporter vector of TGIF2-3`UTR (upper image) and various amounts of miR-210-5p mimic, and dual-luciferase activity was measured 30 h later. (C-D) HOS cells were transfected with Flag-TGIF2-3`UTR vector and various amounts of miR-210-5p mimic. Flag-TGIF2 protein levels were detected by western blot analysis and the relative Flag-TGIF2 levels were estimated by densitometry; the ratios were calculated relative to the actin level. The expression of transfected miR-210-5p was confirmed by qPCR. (E-F) Similar experiments to (C-D) were performed in MT4 cells. The data are presented as mean ± SD for three biological replicates, and statistical significance compared to the controls was calculated by *t* test. *P < 0.05, **P < 0.01, ***P < 0.001, NS: not significant.

It is known that miRNAs mainly act by post-transcriptionally regulating target gene expression. To predict the target genes of miR-210-5p, we identified the common targets predicted by three platforms (PicTar, TargetScan, and miRanda), which produced 14 targets. We then used FINDTAR3 and RNA22 software to predict the potential miR-210-5p-binding sites within the 3`untranslated region (3`UTR) of these predicted targets. The results suggested that TGIF2 might be a target of miR-210-5p (Fig 2A). To confirm the results of the bioinformatics analysis, we constructed a reporter plasmid by cloning the TGIF2 3`UTR into the 3`UTR of firefly luciferase. After co-transfecting this reporter plasmid and miR-210-5p mimics into HOS cells, we observed that miR-210-5p mimics decreased the luciferase expression in a dose-dependent manner (Fig 2B). To confirm whether the effect of miR-210-5p on TGIF2 involves post-transcriptional regulation, an expression vector encoding Flag-TGIF2-3`UTR fusion protein was constructed, in which the open reading frame of TGIF2 was followed by its 3`UTR. This expression vector was then co-transfected with miR-210-5p mimics into HOS cells, and Flag-TGIF2 and miR-210-5p expression were detected. Paralleling the previous results, the miR-210-5p mimics downregulated the Flag-TGIF2 protein level in a dose-dependent manner (Fig 2C). Furthermore, we overexpressed the miR-210-5p mimic in MT4 cells, and western blot analysis showed that the endogenous TGIF2 was specifically suppressed (Fig 2E). The amount of miR-210-5p mimic transfected was confirmed by qPCR in HOS and MT4 cells (Fig 2D–2F). Meanwhile, we have confirmed that miR-210-5p mimic negative control (NC) did not affect the TGIF2 expression (S3 Fig). These results showed that miR-210-5p downregulated TGIF2 by targeting the 3`UTR of TGIF2.

### HIV-1 infection downregulates TGIF2 via miR-210-5p

**Fig 3:**
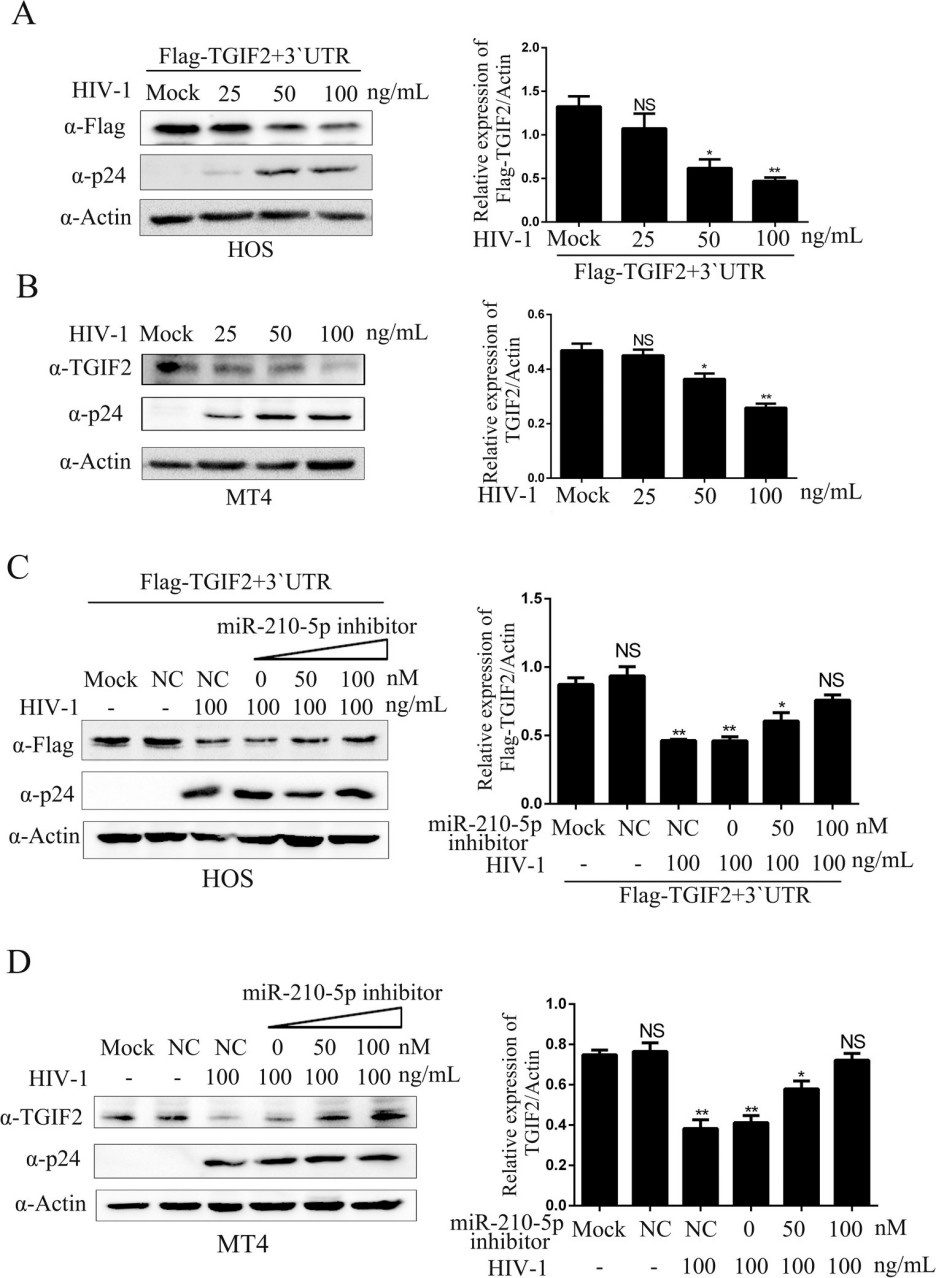
Effects of HIV-1 infection on TGIF2. (A) HOS cells were inoculated with various amounts of HIV-1 and transfected with Flag-TGIF2-3`UTR vector for 30 h. Flag-TGIF2 expression was analyzed by western blot analysis. (B) MT4 cells were inoculated with various amounts of HIV-1, and endogenous TGIF2 expression was analyzed by western blot analysis. (C) HOS cells were transfected with miR-210-5p inhibitor and Flag-TGIF2-3`UTR vector followed by HIV-1 inoculation as indicated. (D) MT4 cells were transfected with miR-210-5p inhibitor followed by HIV-1 inoculation as indicated. The viral p24 protein was detected with anti-p24 antibody. In all experiments, the relative Flag-TGIF2 or TGIF2 levels were estimated by densitometry; the ratios were calculated relative to the actin control. The data are presented as mean ± SD for three biological replicates, and statistical significance compared to the controls was calculated by *t* test *P < 0.05, **P < 0.01, NS: not significant.

To elucidate whether HIV-1 infection downregulates TGIF2, HOS cells transfected with the Flag-TGIF2-3`UTR vector were inoculated with various concentrations of HIV-1. Western blot analysis showed that Flag-TGIF2-3`UTR was markedly downregulated in a virus dose-dependent manner (Fig 3A). Moreover, the endogenous TGIF2 in HIV-1-inoculated MT4 cells was downregulated in a virus dose-dependent manner (Fig 3B). To further explore whether HIV-1-induced TGIF2 downregulation involved the upregulation of miR-210-5p, a miR-210-5p inhibitor, which could increase TGIF2 expression (S4 Fig), was co-transfected with Flag-TGIF2 into HOS cells (Fig 3C) or transfected into MT4 cells (Fig 3D) after HIV-1 incubation for 12 h. Western blot analysis showed that the TGIF2 downregulation caused by HIV-1 was almost eliminated in the miR-210-5p inhibitor-transfected cells but not in the NC inhibitor- and mock-transfected cells (Fig 3C and 3D). These results clearly demonstrated that HIV-1 infection downregulated TGIF2 by increasing miR-210-5p expression.

### TGIF2 reduced the Vpr-induced ratio of G2/M phase cells

**Fig 4:**
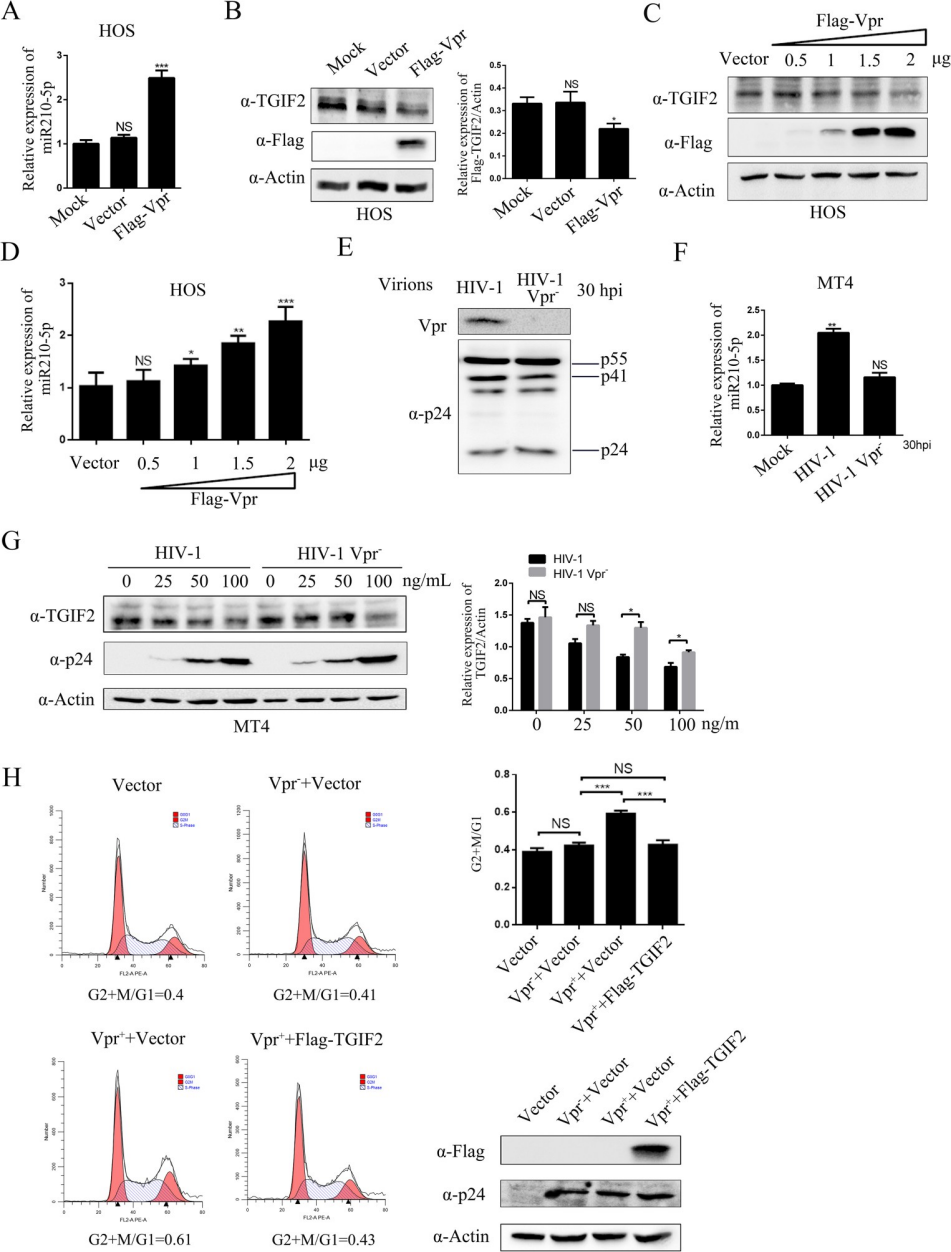
Effect of Vpr-induced TGIF2 downregulation on the cell cycle. (A-B) HOS cells were transfected with Flag-Vpr for 30 h. miR-210-5p and TGIF2 expression was determined by qPCR and western blot analysis, respectively. (C-D) HOS cells were transfected with Flag-Vpr for 30 h in 6-well cell plates. miR-210-5p and TGIF2 expression was determined by qPCR and western blot analysis, respectively. (E) pNL4-3.Luc.R+E- (containing the full-length vpr gene) or pNL4-3.Luc.R-E- (which cannot express Vpr protein) and VSV-G were transfected into HEK293T cells for 48 h. Virions collected from the culture media (using virus precipitation solution) were analyzed by western blot analysis with anti-Vpr antibody. (F) MT4 cells were inoculated with HIV-1 or HIV-1 Vpr- virus for 30 h, and miR-210-5p expression was detected by qPCR. (G) MT4 cells were inoculated with HIV-1 or HIV-1 Vpr^-^ virus at various concentrations and TGIF2 expression was detected by western blot analysis. (H) HOS cells overexpressing TGIF2 were inoculated with HIV-1 or HIV-1 Vpr^-^ virus for 48 h and the cell cycle was analyzed by flow cytometry. The percentages of G1 and G2+M cells were determined using ModFit software and the G2+M/G1 ratios were analyzed. Viral infection was confirmed by anti-p24 antibody detection. In all experiments, the relative Flag-TGIF2 or TGIF2 levels were estimated by densitometry; the ratios were calculated relative to the actin control. The data are presented as mean ± SD for three biological replicates, and statistical significance compared to the controls was calculated by *t* test. *P < 0.05, **P < 0.01, ***P < 0.001, NS: not significant.

Next, we tried to determine which, if any, of the HIV-1 proteins regulate miR-210-5p expression. HOS cells were co-transfected with plasmids expressing each of the HIV proteins (Tat, Gp120, Env, Nef, Gag, Pol, Vif, Vpu, Rev, or Vpr) and the putative promoter region of miR-210 (miPPR-210) with either the 2300-bp full promoter of miR-210-5p or the 894-bp core promoter elements upstream of miR-210-5p. The luciferase activity assay indicated that the Vpr protein stimulated the miR-210-5p promoter activity to the greatest degree (S5 Fig). Further overexpression experiments showed that Vpr protein can upregulate miR-210-5p by >2.5-fold and markedly downregulate the TGIF2 protein (Fig 4A and 4B). To confirm this function of Vpr during HIV-1 infection, we constructed a Vpr-deleted virus (HIV-1-Vpr^-^) (Fig 4C). Compared to HIV-1-Vpr^-^, miR-210-5p was upregulated in MT4 cells inoculated with wildtype HIV-1 (Fig 4D). Additionally, TGIF2 downregulation was extremely weakened in MT4 cells inoculated with HIV-1 Vpr^-^ compared to wildtype HIV-1 (Fig 4E). Moreover, it has been reported that TGIF2 promotes cancer cell proliferation, and the ability of Vpr to induce G2 arrest is well known. Thus, we wondered whether downregulated TGIF2 was associated with Vpr-induced G2/M arrest. HOS cells overexpressing TGIF2 were inoculated with HIV-1 or HIV-1 Vpr^-^, and flow cytometry was used to analyze the cell cycle. The results showed that TGIF2 reduced HIV-1-induced G2/M arrest to the level in the mock-inoculated or HIV-1 Vpr^—^inoculated cells (Fig 4F). These results indicated that Vpr induced G2/M arrest by upregulating miR-210-5p to downregulate TGIF2 protein.

### miR-210-5p is regulated by Vpr via NF-κB p50

**Fig 5:**
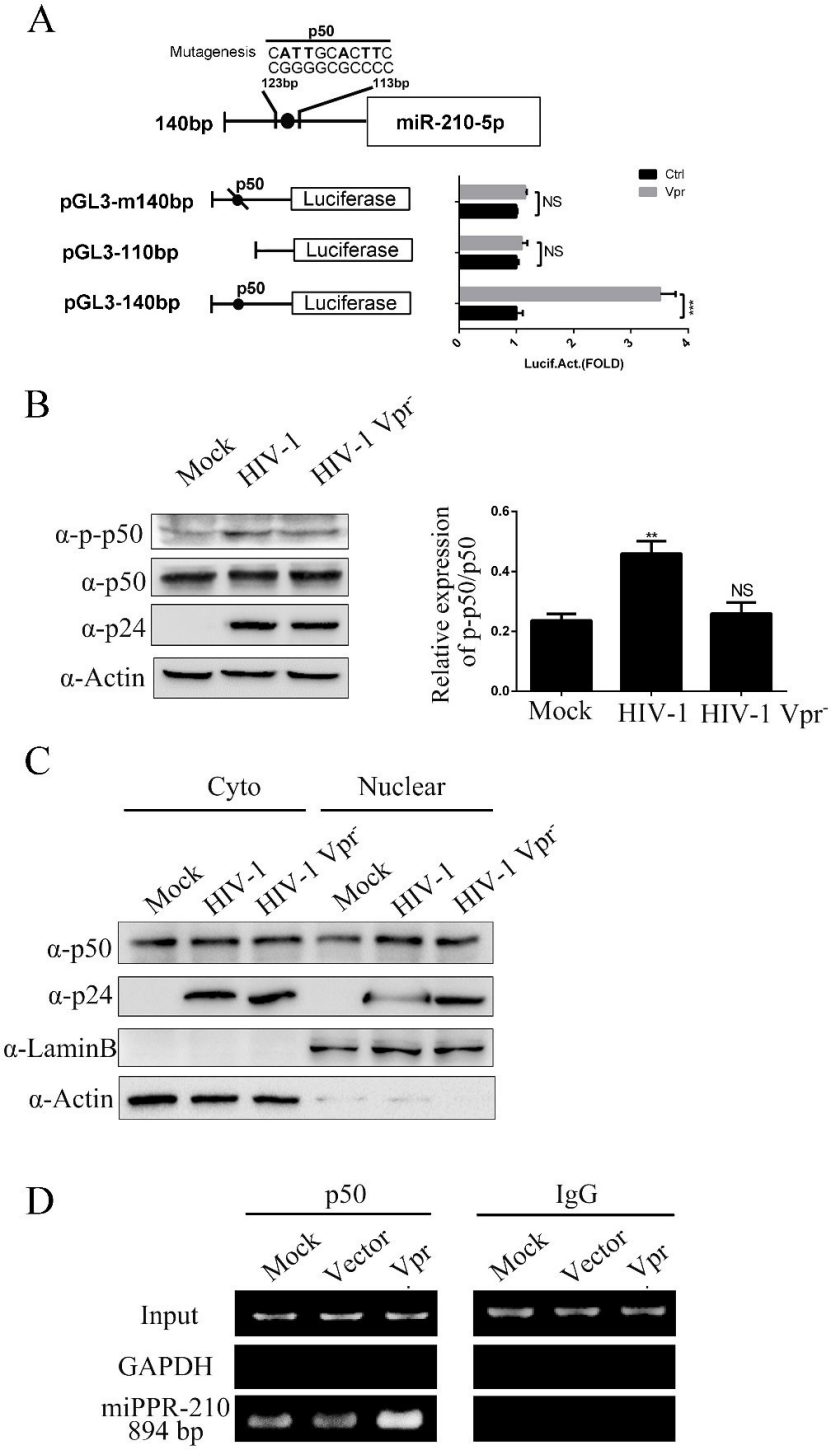
Vpr-mediated regulation of miR-210-5p expression. (A) Promoter analysis using a series of reporter plasmids involving the miR-210-5p promoter (intact, f1; truncated, f2; mutated, f3) with pCMV-Flag-Vpr in HOS cells. (B) p50 and p-p50 protein levels were determined by western blot analysis in MT4 cells inoculated with HIV-1 or HIV-1 Vpr^-^ virus. The relative p-p50 or p50 levels were estimated by densitometry; the ratios of p-p50 were calculated relative to p50. (C) Nucleus-p50 (N-p50) or cytoplasm-p50 (C-p50) protein levels were determined by western blot analysis in MT4 cells inoculated with HIV-1 or HIV-1 Vpr^-^ virus. The relative N-p50 or C-p50 levels were estimated by densitometry; the ratios of N-p50 were calculated relative to C-p50. (D) HOS cells were transfected with vector or Vpr for 30 h and were assessed by ChIP assay, as described in the Materials and Methods. The data are presented as mean ± SD for three biological replicates, and statistical significance compared to the controls was calculated by *t* test. **P < 0.01, ***P < 0.001, NS: not significant.

Several previously reported conserved regulatory elements were found in the consensus sequence of the putative promoter region of miR-210 (miPPR-210), including binding sites for NF-κB p50 [27, 28]. To analyze the role of NF-κB p50 in HIV-1-induced miR-210-5p expression, we constructed a series of reporter plasmids (intact, f1; truncated, f2; mutated, f3) in which either the normal miR-210-5p promoter or mutants were fused to the 5`end of the firefly luciferase gene (Fig 5A). These reporter plasmids were co-transfected with Vpr into HOS cells. Normal miR-210-5p displayed clear Vpr inducibility, but this was decreased by the promoter truncation mutant, indicating that the p50 binding site was involved in the induction of miR-210-5p by Vpr. Thereafter, MT4 cells were inoculated with HIV-1 or HIV-1 Vpr^-^ virus, and the p50 and p-P50 protein levels were determined by western blot analysis. The results indicated that Vpr promoted p50 activity (Fig 5B). It is well known that NF-κB p50 regulates gene transcription when it translocates from the cytoplasm to the nucleus. Therefore, we detected p50 expression in the nucleus and cytoplasm after inoculating MT4 cells with HIV-1 or HIV-1 Vpr^-^ virus. The result showed that p50 expression in the nucleus was obviously increased in HIV-1-inoculated cells compared to in mock-inoculated cells or cells inoculated with HIV-1 Vpr- (Fig 5C). Moreover, ChIP assays were used to further verify that Vpr induced NF-κB p50 to regulate the miR-210-5p promoter (Fig 5D). Together, these results demonstrated that the HIV-1 Vpr protein stimulated miR-210-5p expression via NF-κB p50.

## Discussion

During a viral infection, a variety of intracellular events generate responses in infected cells. This host response represents the first line of defense against viral infection. However, viruses adopt various strategies to evade these host defenses. Accumulating evidence indicates that miRNAs of both viral and host origin may influence the host-virus interactions in a variety of ways: as direct modulators of viral replication, as factors affecting viral susceptibility, and as indirect modulators of cellular genes that influence viral propagation and cell proliferation. In this study, we investigated the role of miRNAs in HIV-1 infection and found that HIV-1 infection upregulated miR-210-5p in order to suppress TGIF2, which resulted in G2 arrest. In addition, our data indicated that Vpr upregulates miR-210-5p level by activating NF-κB p50.

First, we found that HIV-1 appears to enhance the expression of several miRNAs. Specifically, miR-210-5p was upregulated in HIV-1-inoculated cells and HIV-infected individuals, which we considered might be mediated by the Vpr protein. miR-210 (initially we had not determined whether miR-210-3p or miR-210-5p was involved) is upregulated in several cancers and is significantly associated with poor clinical outcomes [29–31]. Although the mechanism has not been previously studied, it has been reported that miR-210 expression is changed in HIV-1-infected individuals and is associated with markers of systemic inflammation [32]. Moreover, studies have also reported that type A influenza virus and cytomegalovirus (CMV) infection can dysregulate miR-210 expression [33, 34]. Furthermore, in hepatitis B virus (HBV)-related hepatocellular carcinoma (HCC) tissues, miR-210-3p was significantly upregulated and its target genes were downregulated in HBV-positive HCC cells [35]. However, a study found miR-210 to be expressing in human red blood cells, but not in the exosome and serum of the same patient [36]. Another study showed that miR-210 only have expression in CD8^+^ T cells derived from long-term non-progressors after HIV-1 infection [37]. Moreover, recently a report indicated that miRNA-210-5p was up-regulated during Latent HIV infection of the resting T cells [38]. In our study, serum *in-vivo* results from HIV-infected individuals and T cells *in-vitro* results from MT4 cells and Jurkat cells infected with HIV-1 confirmed the changes in miR-210-5p expression. These studies indicated that miR-210 might broadly involve in interaction between HIV-1 and host and play different functions in different period during HIV infection by regulating its targeted genes.

A review about miR-210 indicated that miR-210 was involved in numerous biological processes almost throughout the human body, including angiogenesis, the DNA damage response, cell proliferation, and apoptosis [39]. Therefore, we searched the targeted gene of miR-210-5p during HIV-1 infection and found that TGIF2 is a target of miR-210-5p and was regulated during HIV-1 infection. TGIF2 is a transcription regulator that plays essential roles in the regulation of development and cell fate decisions. Previous research indicated that upregulated TGIF2 was restrained by restoration of miR-34c expression in HCC, while miR-148a interacted with TGIF2 and thereby moderated ovarian cancer cell proliferation and invasion [40, 41]. Moreover, TGIF2 has been recognized as an effective regulator in many other cancer types. TGIF2 induced the growth of glioma cells by promoting the cell cycle and inhibiting apoptosis [42]. In non-small-cell lung cancer, TGIF2 contributed to cell proliferation, migration, invasion, and epithelial-mesenchymal transition [43]. Our study demonstrated that TGIF2 was regulated by HIV-1 and played a role in the virulence process. Vpr induced TGIF2 downregulation, thereby causing G2 arrest, which might help virus replication and induce pathological changes. These findings support the hypothesis that cellular miRNAs play important roles during virus infections and modulate the host-pathogen interactions.

HIV-1 infection regulates miRNA expression mainly via viral accessory proteins. The accessory protein Nef upregulates miR-718, which targets PTEN in order to activate the AKT/mTOR pathway [44]. Tat, another accessory protein of HIV-1, promotes astrocyte activation by inducing miR-34a and miR-138 expression and thereby downregulating SIRT1 [45]. Vpr was also reported to induce miR-122, which increases hepatitis C virus (HCV) replication, and to induce miR-942-5p, which affects Kaposi’s sarcoma-associated herpesvirus [46, 47]. HIV-1 infection involves a complex regulatory process. The most studied function of Vpr is the induction of G2 arrest, which can increase HIV-1 replication by affecting the long terminal repeats (LTR) and virion production, and it can also induce HIV-1-infected T cell apoptosis, destroying the immune system. Although Vpr can directly induce G2 arrest through several proteins, in this study, we identified a novel miRNA-related mechanism by which Vpr regulates the cell cycle. This mechanism represents a good complement to direct regulation. Moreover, Vpr is found both in virions and as free molecules in the plasma of HIV-infected patients [48], and miRNAs can be packaged into exosomes and easily secreted by cells to affect other cells. As we found that miR-210-5p was increased in the serum of HIV-1-infected individuals compared to healthy individuals, this indicates that the induction of miR210-5p by Vpr might not only affect infected cells but also non-infected cells.

We also determined the mechanism of Vpr-induced miR-210-5p upregulation. Our results showed that Vpr enhanced p50 activity by increasing the p-p50 level, which can bind to the UTR of miR210-5p to promote its expression. p50 is an NF-κB transcription factor involved in several cellular functions such as cell proliferation, inflammation, and the innate immune response [49, 50]. It has recently been reported that NF-κB is induced during various viral infections, including HBV, HCV, Epstein-Barr virus, and herpes simplex virus type 1 infections, and that this induction promotes viral replication [51]. Tat protein can induce miR-222 expression by increasing the transcriptional activity of NF-κB upstream of the miR-222 promoter [52]. Studies have shown that Vpr activates the NF-κB pathway as well as enhancing transcription from the HIV-1 LTR promoter [53, 54]. Further studies have demonstrated that Vpr activates the NF-κB pathway by modulating the phosphorylation of p65 and p100 to promote G2 arrest [55, 56]. Phosphorylation also regulates the binding ability of NF-κB p50 and influences NF-κB mediated transcription. In this study, we first found that Vpr stimulates the NF-κB pathway by modulating the phosphorylation of p50. However, it is necessary to further research the molecular mechanism behind this function of Vpr regarding p50 activation.

In conclusion, we have identified an HIV-1 virus-host interaction wherein Vpr enhances the activity of p50, which results in miR-210-5p upregulation and then TGIF2 suppression. This pathway might be one of the mechanisms underlying Vpr-induced G2 arrest, and it reveals a new interaction between HIV-1 and its host. Given the pleiotropic effects of Vpr on host cells, this has implications for the pathology of this important virus. These data provide new insights into the interactions between viral proteins and host cells whereby Vpr induces G2 arrest by enhancing miR-210-5p.

## Supporting information

S1 Fig Determination of several miRNAs expression during HIV-1 infection. MT4 cells were inoculated with HIV-1 for 48 h. Several miRNAs expression was measured with qPCR. The ratios were calculated relative to the mock infection.
(TIF)

S2 Fig Determination of miR-210-5p expression during HIV-1 infection in Jurkat cells. Jurkat cells were inoculated with HIV-1 for 48 h at various concentrations. miR-210-5p expression was measured with qPCR. The data are presented as mean ± SD for three biological replicates, and statistical significance compared to the controls was calculated by *t* test. *P < 0.05, **P < 0.01, ***P < 0.001, NS: not significant.
(TIF)

S3 Fig The effect of miR-210-5p mimic NC to TGIF2. (A) HOS cells were transfected with the luciferase reporter vector of TGIF2-3`UTR and various amounts of miR-210-5p mimic NC, and dual-luciferase activity was measured 30 h later. (B) MT4 cells were transfected with various amounts of miR-210-5p mimic NC, and TGIF2 was measured. The data are presented as mean ± SD for three biological replicates, and statistical significance compared to the controls was calculated by *t* test. NS: not significant.
(TIF)

S4 Fig The effect of miR-210-5p inhibitor to TGIF2. (A) HOS cells were transfected with the Flag-TGIF2-3`UTR and various amounts of miR-210-5p inhibitor, and Flag-TGIF2-3`UTR was measured. (B) MT4 cells were transfected with various amounts of miR-210-5p inhibitor, and TGIF2 was measured. The data are presented as mean ± SD for three biological replicates, and statistical significance compared to the controls was calculated by *t* test. *P < 0.05, **P < 0.01, ***P < 0.001, NS: not significant.
(TIF)

S5 Fig The effect of HIV-1 proteins to the miR-210-5p promoter. HOS cells were co-transfected with miR-210-full-promoter-luciferase reporter plasmid (miPPR-210 2.3K) or miR-210-core-promoter-luciferase reporter plasmid (miPPR-210 894bp) and the plasmids encoding each of 10 HIV-1 proteins, as indicated, for 30h. The activity of miR-210 promoter was measured by luciferase activity assays.
(TIF)

S1 Table Primers and oligonucleotide sequences in the study. (DOCX)

S2 Table Differentially expressed miRNAs in microassay. (XLS)

S1 Raw images (RAR)
