# Supplementary figures and images for: HIV-1 Vpr protein upregulates microRNA-210-5p expression to induce G2 arrest by targeting TGIF2
Source: PLoS One. 2021 Dec 29;16(12):e0261971. doi: 10.1371/journal.pone.0261971 (PMC8716043; doi:10.1371/journal.pone.0261971)

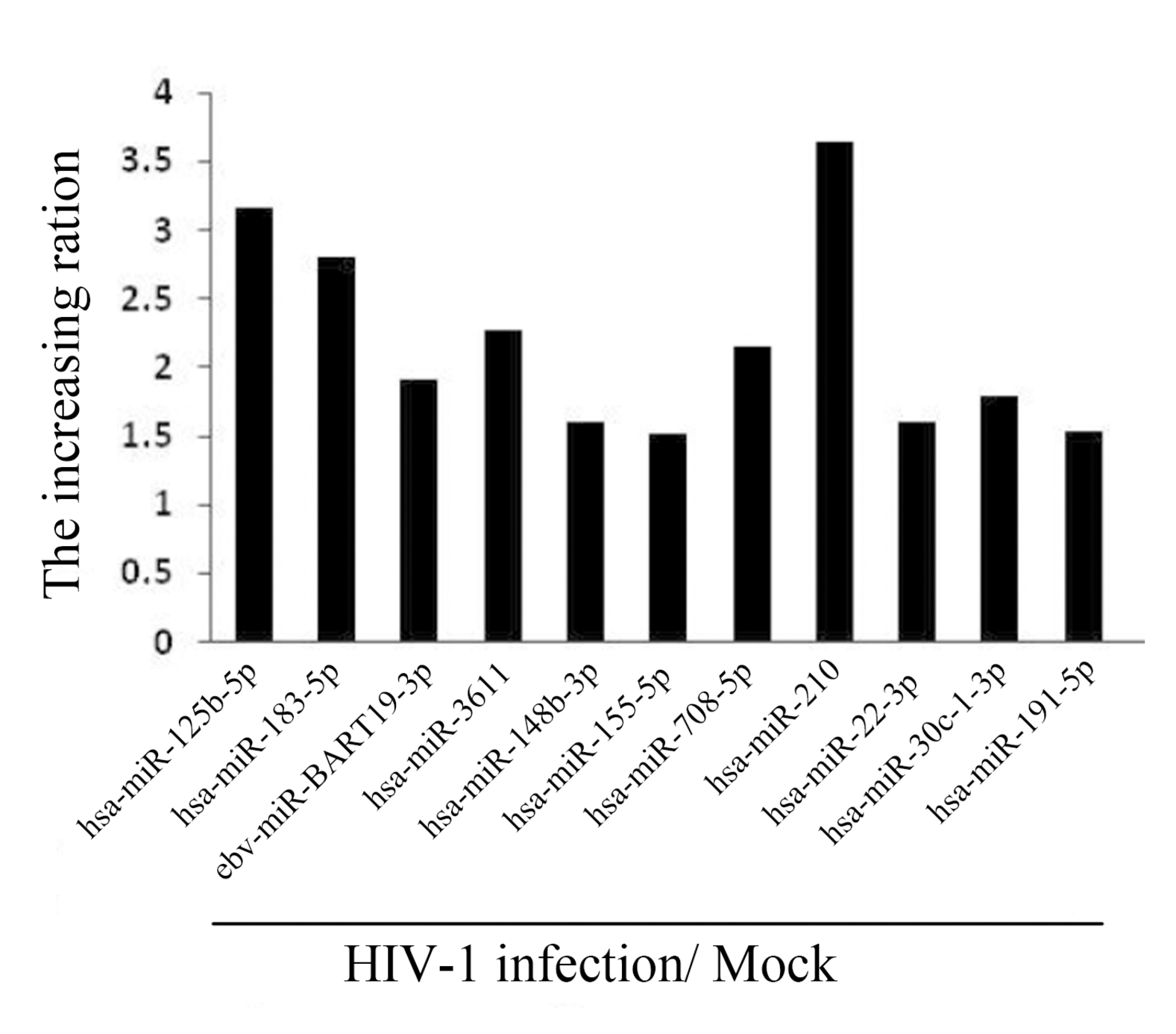

Supplement: S1 Fig — MT4 cells were inoculated with HIV-1 for 48 h. Several miRNAs expression was measured with qPCR. The ratios were calculated relative to the mock infection. (TIF) [file pone.0261971.s001.tif]

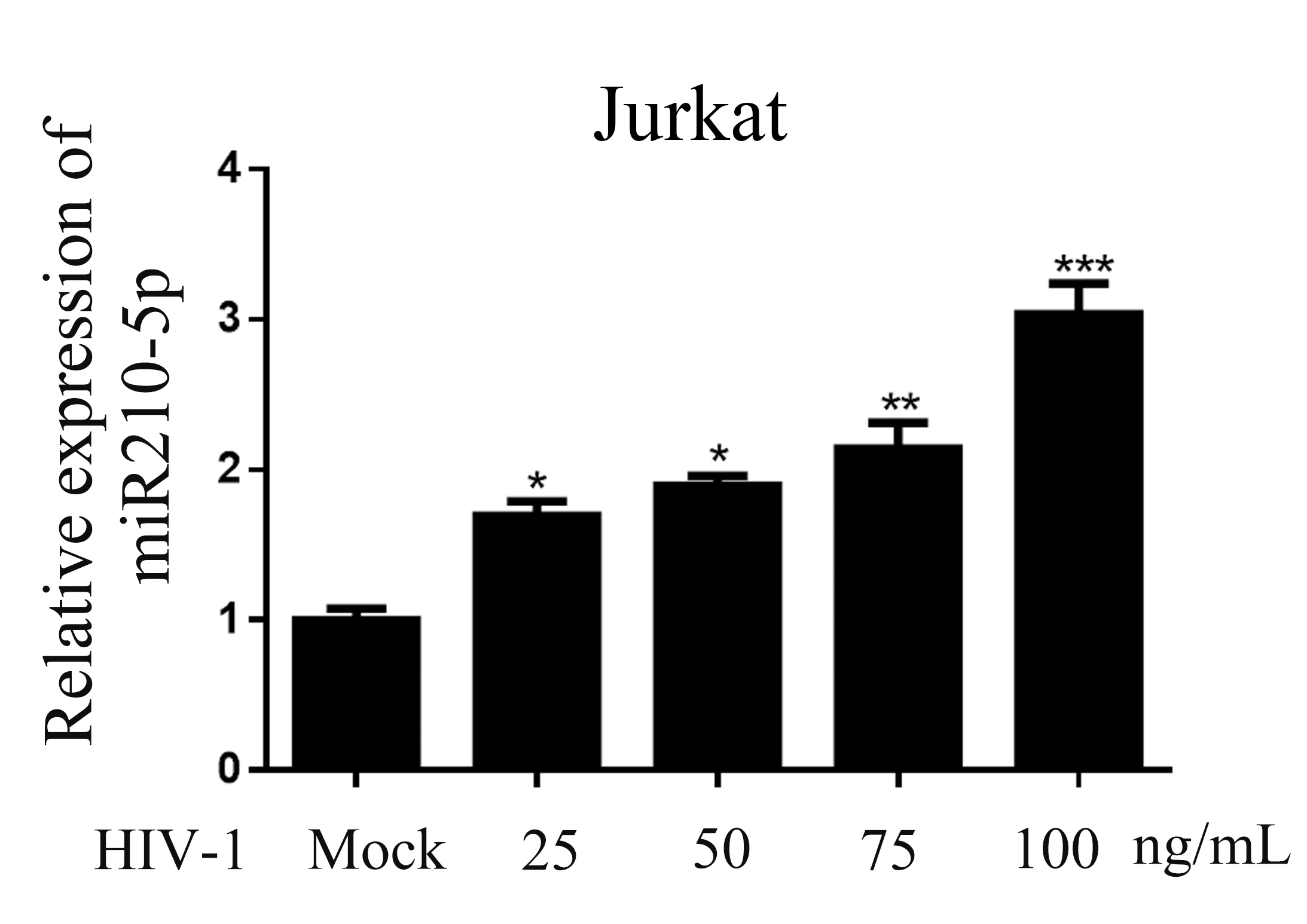

Supplement: S2 Fig — Jurkat cells were inoculated with HIV-1 for 48 h at various concentrations. miR-210-5p expression was measured with qPCR. The data are presented as mean ± SD for three biological replicates, and statistical significance compared to the controls was calculated by t test. *P < 0.05, **P < 0.01, ***P < 0.001, NS: not significant. (TIF) [file pone.0261971.s002.tif]

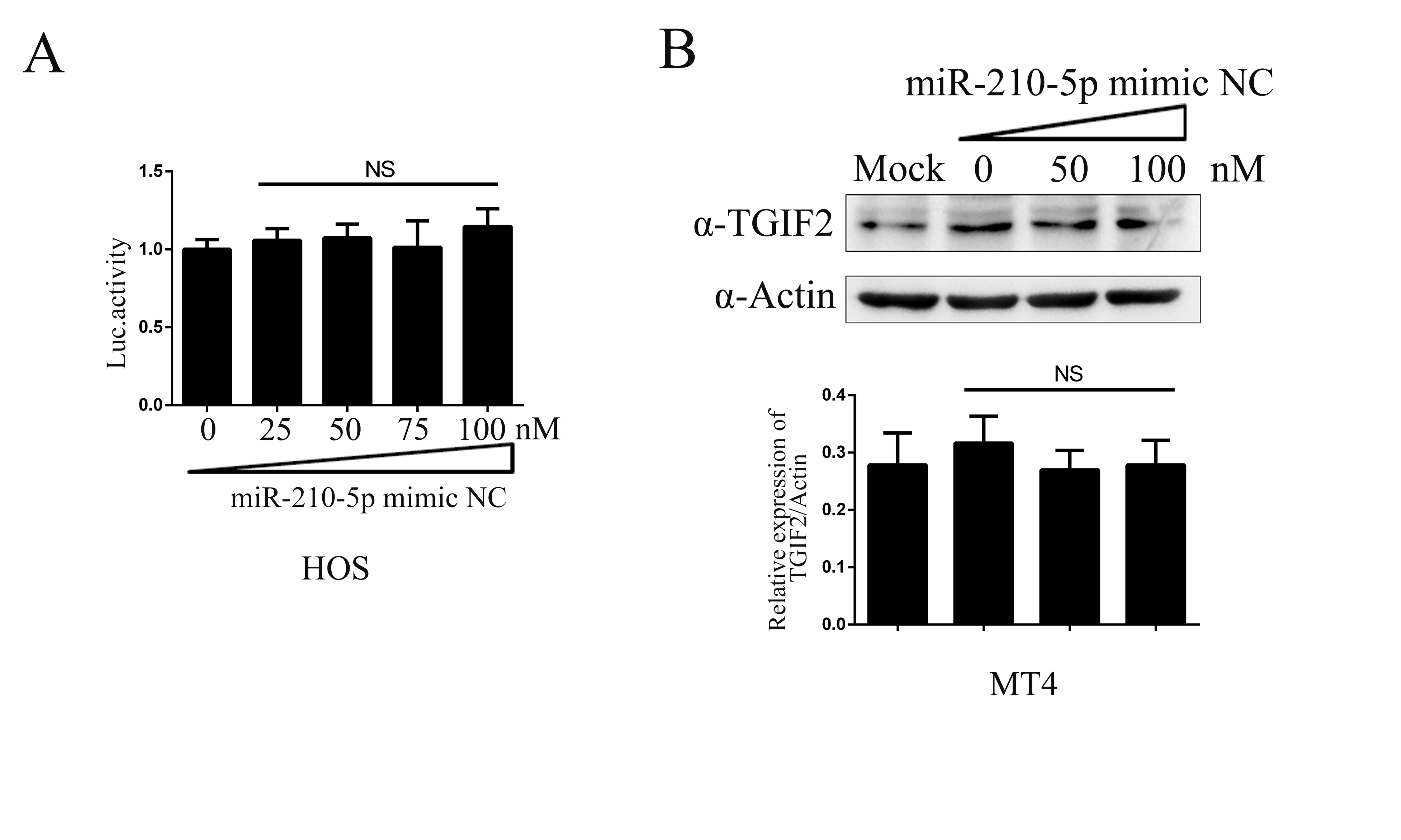

Supplement: S3 Fig — (A) HOS cells were transfected with the luciferase reporter vector of TGIF2-3`UTR and various amounts of miR-210-5p mimic NC, and dual-luciferase activity was measured 30 h later. (B) MT4 cells were transfected with various amounts of miR-210-5p mimic NC, and TGIF2 was measured. The data are presented as mean ± SD for three biological replicates, and statistical significance compared to the controls was calculated by t test. NS: not significant. (TIF) [file pone.0261971.s003.tif]

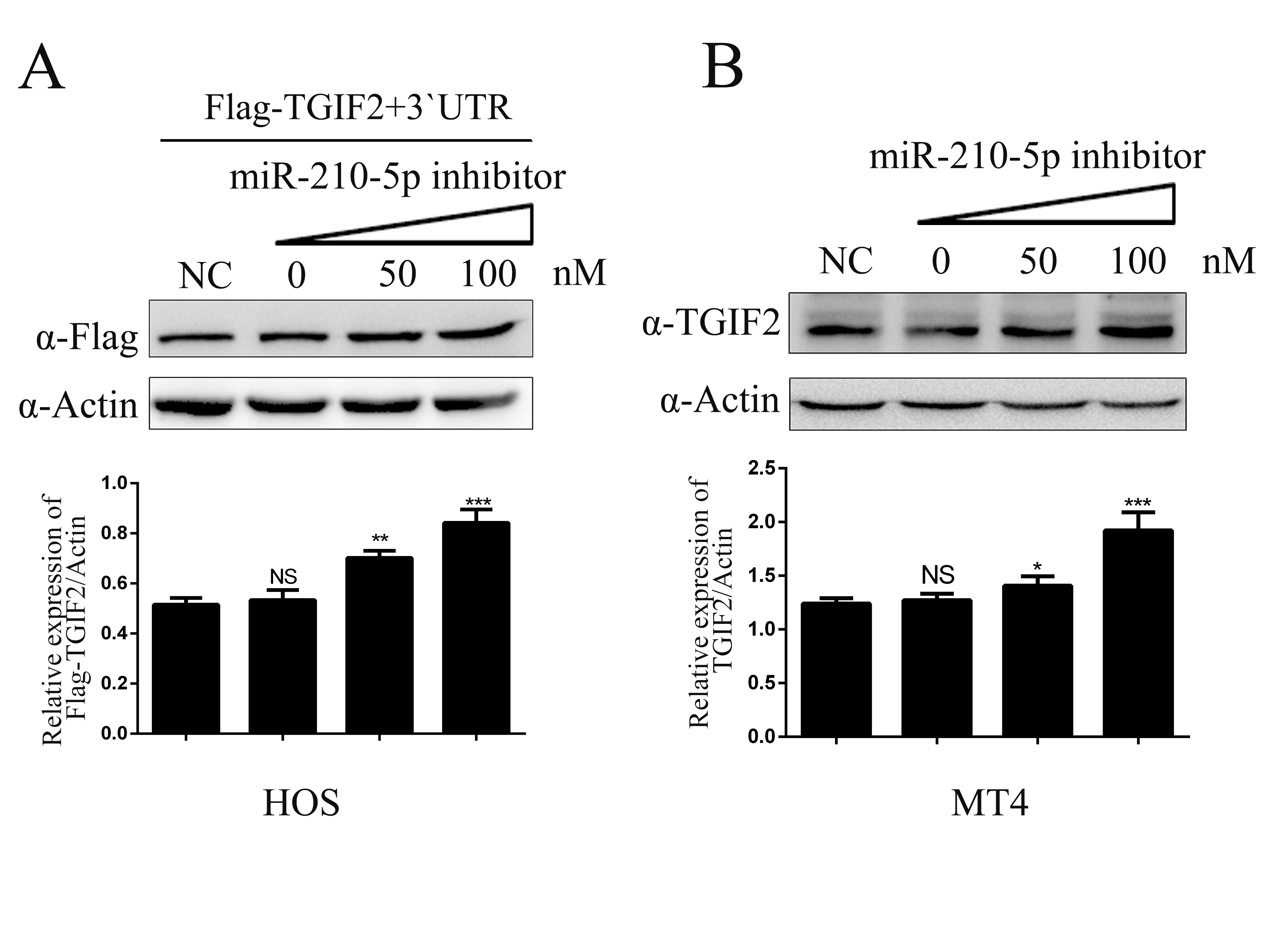

Supplement: S4 Fig — (A) HOS cells were transfected with the Flag-TGIF2-3`UTR and various amounts of miR-210-5p inhibitor, and Flag-TGIF2-3`UTR was measured. (B) MT4 cells were transfected with various amounts of miR-210-5p inhibitor, and TGIF2 was measured. The data are presented as mean ± SD for three biological replicates, and statistical significance compared to the controls was calculated by t test. *P < 0.05, **P < 0.01, ***P < 0.001, NS: not significant. (TIF) [file pone.0261971.s004.tif]

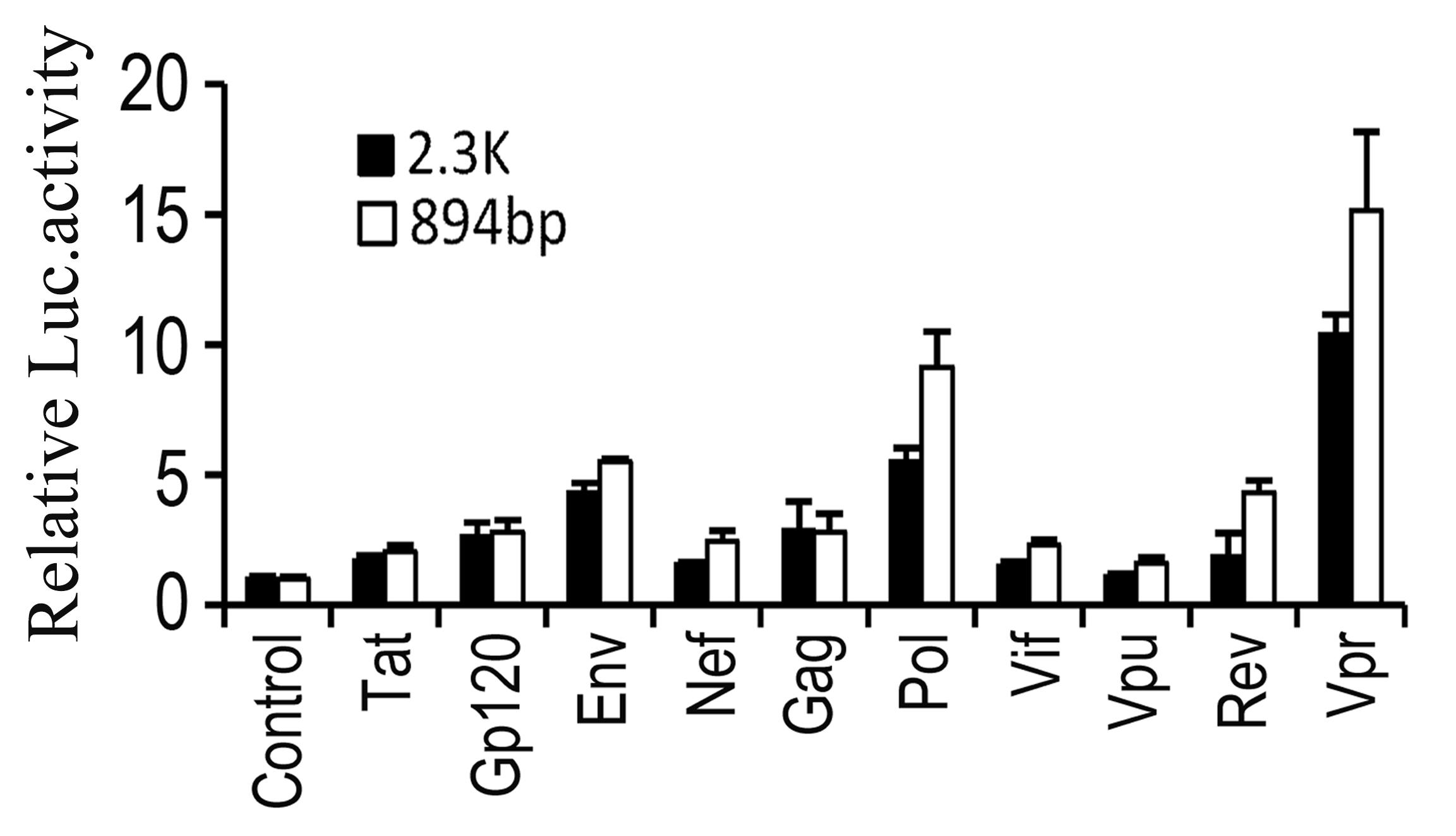

Supplement: S5 Fig — HOS cells were co-transfected with miR-210-full-promoter-luciferase reporter plasmid (miPPR-210 2.3K) or miR-210-core-promoter-luciferase reporter plasmid (miPPR-210 894bp) and the plasmids encoding each of 10 HIV-1 proteins, as indicated, for 30h. The activity of miR-210 promoter was measured by luciferase activity assays. (TIF) [file pone.0261971.s005.tif]
