# Supplementary material for: HIV-1 Vpr protein upregulates microRNA-210-5p expression to induce G2 arrest by targeting TGIF2
Source: PLoS One. 2021 Dec 29;16(12):e0261971. doi: 10.1371/journal.pone.0261971 (PMC8716043; doi:10.1371/journal.pone.0261971)
Supplement: S1 Table — (DOCX) [file pone.0261971.s006.docx]

**Table S1: Primers and** **oligonucleotide sequences in the study**

| **experiment** | **Primer Name** | **Sequence** |
| --- | --- | --- |
| olignucleotide | miR-210-5p mimics | 5’- AGCCCCUGCCCACCGCACACUG -3’ |
|  | miR-210-5p inhibitor  Ctrl mimics  Ctrl inhibitor | 5’- CAGUGUGCGGUGGGCAGGGGCU -3’  5’- UUUGUACUACACAAAAGUACUG-3’  5’- CAGUACUUUUGUGUAGUACAAA-3’ |
| Report assays  (Mutated bases are indicated by underlines) | miPPR-2.3K-F  miPPR-894 bp-F  f1(140bp)-F | 5’- TAATGCATTCTTTTTCCATAAGGATGACGCACA -3’  5’- AATCTCGAGTCGCCAGCAGGACGGA -3’  5’- ATACTCGAGGATCCCAGGTTGGCG -3’ |
|  | f2(110bp)-F  miPPR&f1&f2-R | 5’- ATACTCGAGAGAGGCCGCCCTCCC -3’  5’- TATAAGCTTGGGCGGGCGGAGGGA -3’ |
|  | f3(140bp-mut)-F | 5’- CATTGCACTTCTCAGAGGCCG -3’ |
|  | f3(140bp-mut)-R | 5’- CCCGCCAACCTGGGATC -3’ |
| Primer extension | TGIF2 3`UTR-F: | 5’- CCGCTCGAGGCATCTGCCAAGAAGGGTG -3’ |
|  | TGIF2 3`UTR-R: | 5’- GCGTCGACTCAATGGGACAACTGTGCTTCT -3’ |
|  | TGIF2-F:  TGIF2-R: | 5’- CGGAATTCCGATGTCGGACAGTGATCTAGGTGA-3’  5’- CCGCTCGAGCTACTGGGGATTTTCAGAGACTAAAG -3’ |
